# The Clinical Variables Predicting the Acquisition of Independent Ambulation in the Acute Phase of Stroke: A Retrospective Study

**DOI:** 10.3390/geriatrics8040080

**Published:** 2023-08-11

**Authors:** Masatoshi Koumo, Yoshinori Maki, Akio Goda, Kensaku Uchida, Shohei Ogawa, Tatsumi Matsui, Nozomu Hidemura, Tomohiro Adachi

**Affiliations:** 1 Department of Rehabilitation, Hikari Hospital, Otsu 520-0002, Japan; 2 Department of Rehabilitation, Nishinomiya Kyoritsu Neurosurgical Hospital, Nishinomiya 663-8211, Japan; 3 Department of Neurosurgery, Hikone Chuo Hospital, Hikone 522-0054, Japan; 4 Department of Physical Therapy, Faculty of Health and Medical Sciences, Hokuriku University, Kanazawa 920-1180, Japan

**Keywords:** clinical variables, independent ambulation, acute stroke, unilateral supratentorial stroke lesion, Ability for Basic Movement Scale modified version 2

## Abstract

Background: Predictive factors associated with independent ambulation post-stroke are less commonly reported for patients during the acute phase of stroke. This study aimed to identify the clinical variables predicting ambulation independence in the acute phase of stroke and test the superiority of their prediction accuracy. Methods: Sixty-nine patients, hospitalized in the acute phase for an initial unilateral, supratentorial stroke lesion, were divided into independent (*n* = 24) and dependent ambulation (*n* = 45) groups, with functional ambulation category scores of 4–5 and ≤ 3, respectively. They were evaluated upon admission using the modified Rankin scale (mRS), Stroke Impairment Assessment Set (SIAS) concerning the motor function of the lower extremities, Ability for Basic Movement Scale modified version 2 (ABMS2), and Functional Independence Measure (FIM). Results: The scores of the four measures were significantly different between the groups. A univariate logistic regression analysis identified these variables as prognostic factors for independent ambulation. A receiver operating characteristic curve analysis identified the cutoff values (area under the curve) for the mRS, SIAS, FIM, and ABMS2 as 3 (0.74), 12 (0.73), 55 (0.85), and 23 (0.84), respectively. Conclusion: In summary, the FIM and ABMS2 may be more accurate in predicting ambulation independence in patients with stroke during the acute phase.

## 1. Introduction

Stroke is a global disease experienced by one in four people throughout their life-time. The annual number of people experiencing hemorrhagic or ischemic stroke events globally is approximately 15 million. The incidence of stroke is estimated to increase in the aging society [1]. Stroke is the second-highest cause of death and third-highest cause of disability globally, and is a major cause of bedridden status in patients in Japan [1].

Stroke can result in neurological deficits, including cognitive impairment, unconsciousness, paralysis, aphagia, dysphagia, and impaired ambulation [2]. Rehabilitation therapy for these stroke-related symptoms during the acute and post-acute stroke phases is the gold standard for maintaining and improving the physical and cognitive functions and activities of daily living (ADL) in patients after stroke [3,4,5,6]. In particular, impaired ambulation restricts the ability of patients to perform their ADL after stroke. Therefore, rehabilitation therapy is essential for independent ambulation post-stroke [7].

Predictive clinical factors for independent ambulation have been reported in patients in the convalescent phase of stroke [8,9,10,11]. In clinical practice, some patients in the acute phase of stroke can obtain independent ambulation after rehabilitation therapy. Predicting the acquisition of independent ambulation is important for deciding the destination after acute stroke onset, such as a discharge from or transfer to a hospital. However, to the best of our knowledge, the predictive clinical factors for independent ambulation during the acute phase of stroke remain unclear. Therefore, it is useful to detect the clinical factors affecting the acquisition of independent ambulation during the acute phase of stroke.

This study aimed to identify these predictive clinical variables using clinical scales frequently applied to stroke patients, such as the modified Rankin scale (mRS), the Stroke Impairment Assessment Set (SIAS), the Ability for Basic Movement Scale, modified version 2 (ABMS2), and the Functional Independence Measure (FIM), for the acquisition of independent ambulation in the acute phase of stroke. We also aimed to evaluate the specific cutoff values for each clinical measurement and verify the superiority of their predictive accuracy for gait independence in patients during the acute phase of stroke.

## 2. Materials and Methods

This study enrolled patients with a unilateral supratentorial hemorrhage or infarction, for which rehabilitation therapy was prescribed at the stroke care unit of the Nishinomiya Kyoritsu Neurosurgical Hospital from April to September 2017. The patients underwent computed tomography (CT), magnetic resonance imaging (MRI), and magnetic resonance angiography (MRA) imaging upon admission, and the disease type and foci were identified. Patients were excluded if they had a recurrent stroke episode during admission, a National Institutes of Health Stroke Scale (NIHSS) score of 0 upon admission, an mRS score of 0 upon admission, or a functional ambulation category (FAC) score of 4 upon admission. The database for this study was compiled and maintained by members of the research team using personal computers without internet access.

### 2.1. Clinical Data

The following variables were recorded in the electronic medical records upon admission: age, sex, stroke type, and mRS [12] scores (upon admission and pre-hospitalization). The admission period (days) and discharge destination were recorded upon discharge. The physical function and ADL of the patients were evaluated upon admission and discharge by several physical therapists, using the FAC [13,14], NIHSS [15,16], ABMS2 [17], FIM [18,19], and SIAS concerning the motor function of the lower extremities (SIAS-MLE) [20,21,22], which are widely used in clinical practice. The evaluator read the questions on the evaluation form out loud and verbally confirmed the subject’s answers.

### 2.2. FAC

Based on the ambulation status of patients, the FAC score [13,14] ranges from 0 to 5 as follows: 0 = unable to ambulate, or able to ambulate within parallel bars with support from two or more persons; 1 = requiring continuous support from one person; 2 = requiring intermittent support from one person; 3 = requiring supervision and oral indication from one person; 4 = independent ambulation on a level surface but requiring supervision to negotiate (e.g., stairs, inclines, and nonlevel surfaces); and 5 = completely independent ambulation, including stairs.

### 2.3. mRS

The mRS [12] is a 7-point scale ranging from 0 to 6 that assesses the degree of disability and dependence in daily living for individuals who have experienced a stroke or other neurological disease. The scores are as follows: 0 = no symptoms at all; 1 = no significant disability, but slight symptoms may be present; 2 = slight disability, the patient can perform daily activities independently; 3 = moderate disability, the patient requires some help but can walk without assistance; 4 = moderately severe disability, the patient is unable to walk without assistance and requires substantial help; 5 = severe disability, the patient is bedridden and requires constant nursing care and attention; and 6 = death.

### 2.4. NIHSS

The NIHSS [15,16] is a standardized assessment tool evaluating the severity of neurological deficits in individuals who have experienced a stroke event. It is designed to measure a patient’s neurological status across various domains, including consciousness, language, motor function, and sensory perception. The NIHSS consists of 15 items. Theoretically, its scores can vary from 0 to 40. The severity status of patients can be reflected in a high NIHSS score.

### 2.5. SIAS

The SIAS assesses the impairment of patients post-stroke and has nine subdivisions that measure motor function, muscle tone, sensory function, range of motion, pain, trunk balance, visuospatial ability, speech, and functions on the unaffected side. However, only items concerning the motor function of the lower extremities (SIAS-MLE) [20,21,22] were evaluated in this study, by performing hip-flexion, knee-extension, and foot-pat tests. Each test was scored from 0 to 5 according to the physical performance of the patients post-stroke. The SIAS-MLE theoretically ranges from 0 to 15. Lower scores reflect a lower severity of patient impairment.

### 2.6. FIM

The FIM [18,19] evaluates the ADL of patients and consists of 18 items. Each item is evaluated with a score of 1–7. Lower scores indicate a lower ADL of patients. The motor subscale of the FIM (FIM-motor) theoretically ranges from 13 to 91 and includes 13 items: eating, grooming, bathing, upper body dressing, lower body dressing, toileting, bladder management, bowel management, bed/chair/wheelchair transfer, toilet transfer, tub/shower transfer, walk/wheelchair locomotion, and stairs locomotion. The cognitive subscale of the FIM (FIM-cognitive) theoretically ranges from 5 to 35 and includes five items: comprehension, expression, social interaction, problem solving, and memory.

### 2.7. ABMS2

The ABMS2 scale [17] theoretically ranges from 5 to 30 and evaluates patients’ basic movement ability (sitting and standing balance and trunk ability) using five items: turning over from the supine position, sitting up, remaining sitting, standing up, and remaining standing. Each item is scored from 1 to 6 according to a patient’s ability as follows: 1 = prohibited from moving (because of a medical problem such as unstable vital signs and complications), 2 = totally dependent, 3 = partially dependent, 4 = supervised (movement requiring someone present to provide verbal cues or gestures without physical contact), 5 = independent in a special environment, and 6 = completely independent.

### 2.8. Statistical Analysis

The patients enrolled in this study were classified into two groups: an independent ambulation group (FAC score of 4 or 5 upon discharge) and a dependent ambulation group (FAC score of 3 or less upon discharge) [23]. The Shapiro–Wilk test was used to confirm the normality of each variable. As a preliminary step in the logistic regression analysis, a comparison between the two groups was performed using the independent *t*-test (parametric data), Mann–Whitney’s U test (nonparametric data), and chi-square test (categorical variables) to identify the variables that may affect gait independence. Using the hypothesis that the factors could influence the prediction of ambulation independence in patients with acute stroke, a univariate logistic regression analysis (forced entry method) was used to investigate the significant predictive factors for independent ambulation in patients upon discharge post-stroke. In the unadjusted univariate logistic regression analysis, the dependent variable was whether the patient could ambulate independently (independent ambulation (FAC score of 4 or 5) or relied on dependent ambulation (FAC score ≤ 3) upon discharge). The independent variables were the mRS, NIHSS, SIAS-MLE, FIM-motor, FIM-cognitive, and ABMS2, respectively. In the adjusted univariate logistic regression, age, sex, etiology, and mRS pre-hospitalization were added as covariates to the above. The dependent ambulation group was established as the reference group. Receiver operating characteristic (ROC) curves were used to detect the cutoff points of the variables, for which statistical significance was detected in the univariate logistic regression analysis. The SPSS Statistics software version 24 (IBM Corp Armonk, NY, USA) was used in this study. Statistical significance was defined as a *p*-value of <0.05.

This study was approved by the ethics committee of the Nishinomiya Kyoritsu Neurosurgical Hospital (4 October 2021; approval no. 21-02) and conducted in accordance with the Declaration of Helsinki. Written informed consent was obtained from all the participants.

## 3. Results

A total of 69 patients were enrolled in the study. Of these, 42 patients were discharged home, 21 were transferred for the purpose of continuing their rehabilitation, and 6 were discharged to nursing homes or other facilities. The independent and dependent ambulation groups included 24 (*n* = 18 male, *n* = 6 female) and 45 (*n* = 23 male, *n* = 22 female) patients, respectively (*p* = 0.07) (Table 1). The stroke etiology was cerebral infarction (CI) in 59 patients (diagnoses: atherothrombotic cerebral infarction—17, lacunar infarction—18, cardiogenic cerebral embolism—6, and cerebral infarction—18) and intracerebral hemorrhage (ICH) in 10 patients (diagnoses: thalamic hemorrhage—3, capsular hemorrhage—3, and subcortical hemorrhage—4). The average ages (mean ± standard deviation) of the independent and dependent ambulation groups were 69.5 ± 13.9 and 77.6 ± 11.5 years, respectively (*p* = 0.01). The average admission periods (mean ± standard deviation) of the independent and dependent ambulation groups were 16.8 ± 7.6 and 23.9 ± 15.3 days, respectively (*p* = 0.05). In addition, significant differences between the groups were identified for the mRS scores upon admission and the NIHSS, SIAS-MLE, FIM-motor, FIM-cognitive, and ABMS2 scores upon admission (*p* < 0.01; Table 1).

### 3.1. Univariate Logistic Regression Analysis

The adjusted univariate logistic regression analysis showed that the mRS, SIAS-MLE, FIM-motor, and ABMS2 scores upon admission were significant prognostic indicators of the acquisition of independent ambulation upon discharge (*p* < 0.05; Table 2).

### 3.2. ROC Curve Analysis

**Figure 1:**
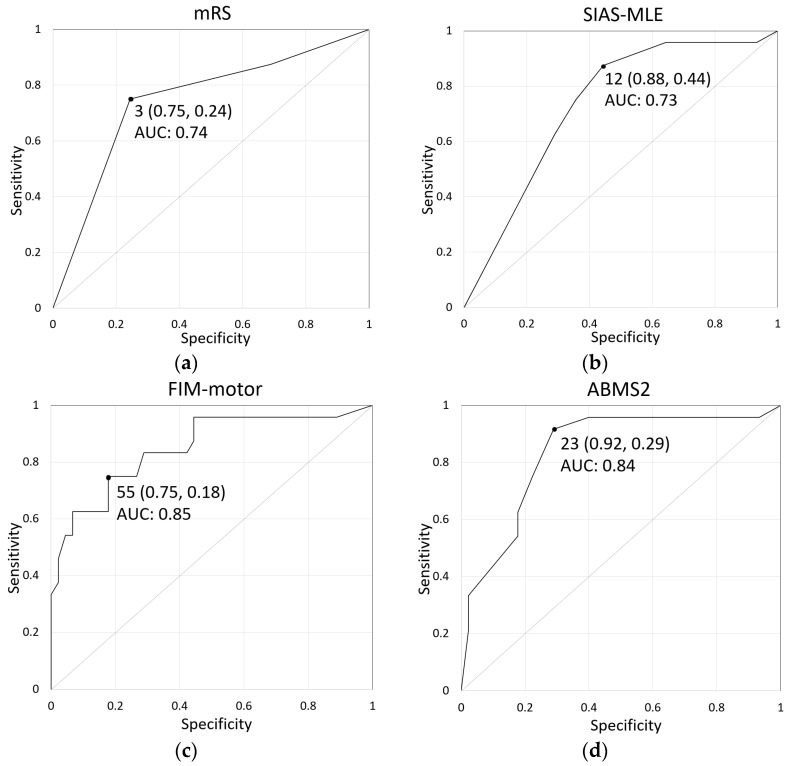
Results of the receiver operating characteristic (ROC) curve analysis. ROC curves show the cutoff values with sensitivity and specificity for each battery ((**a**): mRS, (**b**): SIAS-MLE, (**c**): FIM-motor, and (**d**): ABMS2). mRS, modified Rankin scale; SIAS-MLE, Stroke Impairment Assessment Set—Motor Function of the Lower Extremities; FIM-motor, Functional Independence Measure—motor subscale; ABMS2, Ability for Basic Movement Scale modified version 2; and AUC, area under the curve.

The ROC curves are shown in Figure 1. The cutoff values for the mRS, SIAS-MLE, FIM-motor, and ABMS2 scores upon admission were 3, 12, 55, and 23, respectively. The sensitivity, specificity, and area under the curve for each scale are shown in Table 3.

## 4. Discussion

Our study identified the predictive factors during the acute stroke phase for patients who were not ambulation independent upon hospital admission to achieve independent ambulation (equivalent to an FAC score of 4 or 5) upon discharge. The patients’ baseline characteristics, the severity of the stroke event, and their residual physical and cognitive abilities were crucial for the acquisition of independent ambulation. The mRS, SIAS-MLE, FIM-motor, and ABMS2 scores upon admission were predictive factors for independent ambulation during the acute phase of stroke. The FIM-motor and ABMS2 scores upon admission were significant predictive factors and, as predictive factors related to independent ambulation in the acute phase of stroke, have not previously been examined well; our study is useful in identifying them.

The FAC was reported with an excellent interrater reliability [24]. FAC scores can be correlated with ambulation performance (i.e., speed, step length, and walking distance), measured by a six-minute walking test and ten-meter walking test [24,25]. The dichotomization of FAC scores with a value of four appears to be related to the ambulation ability of stroke patients for a long period after rehabilitation therapy. Although predictive factors in the acquisition of independent ambulation during the acute phase of stroke have not been investigated well, independent ambulation was defined by FAC scores of 4 or 5 in our study [23].

The FIM evaluates patients’ ADL. A correlation between the ADL of patients who experience stroke and their ambulation ability has been described in patients with convalescent stroke [8,9,10,11]. However, this concern has rarely been described in patients in the acute phase of stroke. This is potentially because the management of patients with acute stroke is usually focused on intensive care, and functional recovery through rehabilitation therapy may be less prioritized. Masiero et al. reported that FIM-motor score was correlated with ambulation ability [26]. In their study, the initial FIM-motor score (mean ± standard deviation) for independently ambulating (defined as an FAC score of 3, 4, or 5) patients was 49.67 ± 22.78, the average period (mean ± standard deviation) from the time of stroke onset to discharge from the hospital was 77.9 ± 34.7 days, and the hospital stay length was 51.4 ± 29.5 days [26]. However, this study [26] did not include patients in the acute phase of stroke. 

The utility of ABMS2 scores in predicting the acquisition of independent ambulation has been reported in the acute and convalescent phases of stroke [23,27,28]. Uwatoko et al. analyzed the cutoff value of ABMS2 to predict the acquisition of independent ambulation 14 and 90 days after a stroke [27]. The cutoff values for the ABMS2 score were 26 for independent ambulation 14 days after a stroke event and 15 for independent ambulation on day 90 [27]. Although the mean age of the 67 patients included in their study was slightly higher (73.6 years) [27] than that of the patients enrolled in our study (69.5 years), the cutoff value of 23 for the ABMS2 score in our study seems coherent, as the patients in our study achieved independent ambulation an average of 16.8 days after the stroke event.

The SIAS systematically evaluates impairments related to stroke events; this score is also considered to be reliable for predicting functional outcomes after rehabilitation therapy in patients post-stroke [21,29,30]. In SIAS-MLE, the proximal and distal lower extremity functions are estimated using hip-flexion, knee-extension, and foot-pat tests. Proximal and distal lower extremity variables are highly correlated with ambulation ability [21]. In our study, variables such as SIAS-MLE were evaluated in the enrolled patients. The SIAS-MLE was a predictive factor for independent ambulation with a cutoff score of 12. Ishiwatari et al. reported that the SIAS motor item scores upon admission were significantly higher in an independent ambulation group than in a dependent ambulation group [31]. Their study defined independent ambulation as an FIM gait item score of 6 or 7. In addition, they used the SIAS motor items, which include upper extremity motor function. They did not describe the detailed motor function scores of the patients’ upper and lower extremity motor function [31]. Hiratsuka et al. reported the detailed SIAS-MLE scores of patients independently ambulating (an FAC score of 4 or 5) 15 days after stroke onset [32]. The scores (mean ± standard deviation) were 4.3 ± 1.2, 4.4 ± 1.2, and 4.3 ± 1.0 for the hip-flexion, knee-extension, and foot-pat tests, respectively [32]. Although individual SIAS-MLE scores were not evaluated in our study, the total SIAS-MLE score was similar to that reported by Hiratsuka et al. [32].

Compared to the AUCs of the FIM and ABMS2 scores, those of the mRS and SIAS-MLE appeared inferior in predicting the acquisition of independent ambulation during the acute phase of stroke. The mRS [33] and SIAS-MLE [20] may have been less accurate in capturing subtle differences in disability because of the narrower scaling of the possible scores compared to the other two indices. Therefore, measures of overall functional independence and basic motor skills across multiple domains of ADL (FIM-motor and ABMS2) are likely more accurate in predicting independence in patients with stroke during the acute phase than measures of the degree of impairment in daily living and lower limb motor function impairment upon admission (mRS and SIAS-MLE). To date, there have been no studies that have simultaneously measured these four assessment indices and compared their accuracy in predicting walking independence in acute stroke patients, and this is a novelty of our study.

### Limitations

This study had some limitations. First, this retrospective study was conducted in a single acute stroke hospital, so the sample size is small and requires careful interpretation. To corroborate the findings of the present study, a subsequent study with a larger sample size of approximately 709 people (priori test by G*power (z tests; Logistic regression)) is needed. Second, patients with bilateral stroke lesions, recurrent stroke episodes during admission, infratentorial lesions, or subarachnoid hemorrhage were excluded. Therefore, the findings of this study are not applicable to patients presenting with these symptoms. Third, the enrolled patients were followed up for a short period after stroke occurrence (during the acute care hospital stay only). Thus, our results may not be pertinent for patients followed up over a longer period. Fourth, multiple rehabilitation therapists evaluated the enrolled patients. Therefore, subjective bias cannot be completely excluded. Fifth, we were not able to examine the influence of parameters related to the patient’s ambulatory independence, such as the manner of presentation of the patients, stroke symptoms in the presentation, therapy performed including hospital protocol for stroke management, complications, how many patients required intubation, specific regional deficits, smoking, dyslipidemia, type of stroke, etiology of stroke, type of hospitalization, time of onset of signs and symptoms in relation to admission, door-to-needle time, door-to-puncture time, and recanalization score. Future studies should include these parameters. Sixth, the balance or trunk control abilities that can facilitate independent ambulation in the acute or chronic phase of stroke [31,34,35] were not evaluated in our study. Future research should address the correlation between balance or trunk control abilities and independent ambulation in patients in the acute phase of stroke. Lastly, we analyzed only the clinical scales and scores of the patients in the acute phase of stroke in this study. However, electroencephalography in hyper-acute anterior circulation ischemic stroke and CT perfusion examination in wake-up stroke have been reported to predict outcomes in the acute phase [36,37]. Thus, our study has the limitation that radiological findings were not simultaneously evaluated.

## 5. Conclusions

The present study showed that cutoff values of mRS, SIAS-MLE, FIM-motor, and ABMS2 were associated with the acquisition of independent walking in acute stroke and that the predictive accuracy was higher for FIM-motor and ABMS2 with wider theoretical scales. These cutoff values may be useful for predicting the acquisition of independent ambulation in patients in the acute phase of stroke. Despite some limitations, the findings of this study can help healthcare professionals involved in acute stroke care to predict patient prognoses, design more appropriate rehabilitation schemes, and implement coordinated discharge.

## Figures and Tables

**Table 1:** Baseline characteristics and comparisons of clinical measures post-stroke between the independent and dependent ambulation groups (*n* = 69).

	Independent Ambulation Group (*n* = 24)			Dependent Ambulation Group (*n* = 45)			*p*-Value	Power
Age (y) ^※^	69.5	±	13.9	77.6	±	11.5	0.01	0.70
Sex (male/female) (*n*) ^※※^	18/24 (75%)	/	6/24 (25%)	23/45 (51%)	/	22/45 (49%)	0.07	0.99
Etiology (CI/ICH) (*n*)^※※^	20/24 (83%)	/	4/24 (17%)	39/45 (87%)	/	6/45 (13%)	0.73	0.14
mRS pre-hospitalization (score)	0.3	±	0.6	1.3	±	1.1	<0.01	0.99
Admission period (days)	16.8	±	7.6	23.9	±	15.3	0.05	0.61
Rehabilitation period (days)	15.2	±	7.9	22.3	±	14.8	0.06	0.63
mRS (score)	3.4	±	0.7	4.1	±	0.8	<0.01	0.94
NIHSS (score)	3.1	±	4.8	5.8	±	5.6	<0.01	0.50
SIAS-MLE (score)	13.8	±	3.1	11.2	±	4.2	<0.01	0.76
FIM-motor (score)	60.3	±	14.2	39.0	±	16.5	<0.01	0.99
FIM-cognitive (score)	28.5	±	8.8	20.4	±	9.2	<0.01	0.93
ABMS2 (score)	25.2	±	5.0	18.7	±	6.2	<0.01	0.99

Mann–Whitney U test, ※: independent *t*-test, and ※※: chi-square test. Abbreviations: CI, cerebral infarction; ICH, intracerebral hemorrhage; mRS, modified Rankin scale; NIHSS, National Institutes of Health Stroke Scale; SIAS-MLE, Stroke Impairment Assessment Set—Motor Function of the Lower Extremities; FIM, Functional Independence Measure; and ABMS2, Ability for Basic Movement Scale modified version 2.

**Table 2:** Adjusted and unadjusted univariate regression analyses comparing clinical measures post-stroke for independent vs. dependent ambulation groups, among 69 patients with acute stroke.

	Unadjusted Logistic Regression										Adjusted Logistic Regression									
	B	SE	Wald	df	OR	95% CI			*p*-Value	Power	B	SE	Wald	df	OR	95% CI			*p*-Value	Power

mRS	−1.311	0.412	10.142	1	0.270	0.120	–	0.604	0.001	0.215	−1.127	0.441	6.525	1	0.324	0.136	–	0.769	0.011	0.208
NIHSS	−0.162	0.093	3.013	1	0.851	0.709	–	1.021	0.083	0.922	−0.120	0.084	2.046	1	0.887	0.752	–	1.045	0.153	0.653
SIAS-MLE	0.264	0.119	4.933	1	1.301	1.031	–	1.642	0.026	0.587	0.338	0.138	6.020	1	1.403	1.070	–	1.838	0.014	0.438
FIM-motor	0.102	0.028	12.897	1	1.107	1.047	–	1.170	0.000	0.722	0.092	0.032	8.152	1	1.096	1.029	–	1.167	0.004	0.604
FIM-cognitive	0.102	0.033	9.510	1	1.107	1.038	–	1.181	0.002	0.762	0.062	0.040	2.405	1	1.064	0.984	–	1.150	0.121	0.490
ABMS2	0.261	0.078	11.300	1	1.298	1.115	–	1.512	0.001	0.399	0.256	0.098	6.792	1	1.292	1.066	–	1.566	0.009	0.318

The dependent variables were independent ambulation (FAC score of 4 or 5) or dependent ambulation (FAC score ≤ 3) upon discharge. The dependent ambulation group was established as the reference group. Covariates: age, sex, etiology, and mRS pre-hospitalization. Abbreviations: B, unstandardized coefficient; SE, standard error; df, degrees of freedom; OR, odds ratio; 95% CI, 95% confidence interval; mRS, modified Rankin scale; NIHSS, National Institutes of Health Stroke Scale; SIAS-MLE, Stroke Impairment Assessment Set—Motor Function of the Lower Extremities; FIM, Functional Independence Measure; ABMS2, and Ability for Basic Movement Scale modified version 2; FAC, functional ambulation category.

**Table 3:** Results of the receiver operating characteristic curve analysis among 69 patients with acute stroke.

	Cutoff	Sensitivity	1—Specificity	AUC	95% Confidence Interval	*p*-Value
mRS	3	0.75	0.24	0.74	0.62–0.87	<0.01
SIAS-MLE	12	0.88	0.44	0.73	0.61–0.86	<0.01
FIM-motor	55	0.75	0.18	0.85	0.75–0.95	<0.01
ABMS2	23	0.92	0.29	0.84	0.73–0.94	<0.01

Abbreviations: mRS, modify Rankin scale; SIAS-MLE, Stroke Impairment Assessment Set—the Motor Function of the Lower Extremities; FIM, Functional Independence Measure; ABMS2, Ability for Basic Movement Scale modified version 2; and AUC, area under the curve.

## Data Availability

The data that support the findings of this study are available from the corresponding author, AG, upon reasonable request.
